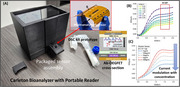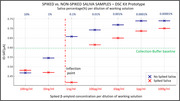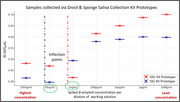# Saliva Sampling and Testing Strategies for Screening AD Biomarkers Using a Portable Antibody Functionalized Bioanalyzer Platform

**DOI:** 10.1002/alz70861_108703

**Published:** 2025-12-23

**Authors:** Srishti Johri, Dylan Layton‐Matthews, Andrew R Frank, Amit Arora, Evgueni Doukhanine, Rafal Iwasiow, Ravi Prakash, Organic Sensors and Devices Lab (OSDL)

**Affiliations:** ^1^ Carleton University, Ottawa, ON Canada; ^2^ Bruyere Health Research Institute, Ottawa, ON Canada; ^3^ DNA Genotek Inc., Ottawa, ON Canada

## Abstract

**Background:**

Saliva collection has been explored for non‐invasive AD biomarker testing in several studies (Kirmess et al.,2021, De Meyer et al.,2020), however, challenges persist in maintaining consistent sampling and reliable benchmarking for the low salivary Aß42 concentration in healthy controls (0.6‐66.1pg/ml; Agnello et al.,2024, Sabbagh et al.,2018). We share preliminary outcomes on using novel saliva collection kit prototypes from DNA Genotek Inc.(Ottawa) for clinical trial deployment of our patent‐pending bioanalyzer platform.

**Method:**

Saliva was collected from healthy control (established using brain MRI at the IMHR, Royal Hospital, Ottawa) using two collection kit prototypes (Drool Saliva Collection(DSC) and Sponge‐tip Saliva Collection(SSC)). Collected samples were spiked to 10 µg/ml of Aß42 and a dilution series (100ng/mL‐100fg/mL) was created using the supernatant. Figure 1(A) shows the portable bioanalyzer (Ab‐OEGFET; Johri et al.,2024, Johri et al.,2025) used for saliva testing.

**Result:**

The Ab‐OEGFET sensor(bioanalyzer) predicts Aß42 concentration through an inversely correlated output current(ID‐SAT). The output (Figure 1B) shows current modulation with gate bias (VSG), and Figure 1(C) shows the current modulation for different concentrations of Aß42(VSG=2.4V).

The human saliva supernatant(HSN) collected via DSC kit prototype was tested first without Aß42 spiking, and then by spiking with 10 µg/mL Aß42, followed by serial dilution. The non‐spiked HSN showed a predictable drop in current with increase in overall protein baseline concentration of saliva, whereas Aß42 spiked HSN showed a clear inflection ∼1ng/ml spiking level (Figure 2). In another experiment (Figure 3), HSN collected from DSC and SSC prototypes were spiked with 10 µg/ml Aß42. The DSC prototype represented inflection in sensing range ∼1ng/ml of spiked protein and the SSC prototype appeared to have inflection point ∼10 ng/ml of spiked protein, showing variation in overall protein baselines from the two collection strategies. As evident, DSC prototype samples had higher background protein concentration whereas samples from the SSC prototype had comparatively higher concentration of dilution buffer.

**Conclusion:**

The saliva sampling prototype testing with bioanalyzer platform was conducted to verify the protein stability and platform capability prior to deployment in an AD dementia clinical trial in Ottawa. The team will now proceed with testing saliva samples from patients with different stages of AD pathology as established using amyloid PET scans (IMHR, Ottawa).